# Mg alloys with antitumor and anticorrosion properties for orthopedic oncology: A review from mechanisms to application strategies

**DOI:** 10.1063/5.0191800

**Published:** 2024-04-17

**Authors:** Zhensheng Lin, Yuhe Wei, Huazhe Yang

**Affiliations:** 1 Medical Engineering Center, Hunan Provincial People's Hospital, The First Affiliated Hospital of Hunan Normal University, Changsha 410005, Hunan, China; 2 Department of Medical Equipment, Tianjin Chest Hospital, Tianjin 300350, China; 3 School of Intelligent Medicine, China Medical University, Shenyang 110122, China

## Abstract

As a primary malignant bone cancer, osteosarcoma (OS) poses a great threat to human health and is still a huge challenge for clinicians. At present, surgical resection is the main treatment strategy for OS. However, surgical intervention will result in a large bone defect, and some tumor cells remaining around the excised bone tissue often lead to the recurrence and metastasis of OS. Biomedical Mg-based materials have been widely employed as orthopedic implants in bone defect reconstruction, and, especially, they can eradicate the residual OS cells due to the antitumor activities of their degradation products. Nevertheless, the fast corrosion rate of Mg alloys has greatly limited their application scope in the biomedical field, and the improvement of the corrosion resistance will impair the antitumor effects, which mainly arise from their rapid corrosion. Hence, it is vital to balance the corrosion resistance and the antitumor activities of Mg alloys. The presented review systematically discussed the potential antitumor mechanisms of three corrosion products of Mg alloys. Moreover, several strategies to simultaneously enhance the anticorrosion properties and antitumor effects of Mg alloys were also proposed.

## I. INTRODUCTION

Primary malignant bone tumor can be classified into several types, including osteosarcoma (OS), chondrosarcoma, Ewing's sarcoma, etc., among which OS is the most common malignant bone tumor that usually occurs in children and adolescent.^1,2^ OS typically appears in epiphyses of long bones such as the proximal tibia, distal femur, and proximal humerus, with clinical symptoms of local swelling, severe pain, impaired joint mobility, and even bone fractures.^3–5^ Osteosarcoma poses a huge threat to human health, and the treatment of which is still challenging due to its high metastatic ability and rapid progression.^6,7^ At present, the principal treatment of OS is surgical intervention to remove the infected bone tissues combined with chemotherapy to eradicate residual tumor cells.^8–10^ However, the drug resistance and serious organ damage of chemotherapy often lead to poor therapeutic effects.^11,12^ After tumor resection, extensive bone defects occur, which cannot be repaired by the bone tissue itself.^13^ The osteosarcoma cells (OCs) remaining around the bone defects can proliferate over days, leading to the recurrence and metastasis of OS.^14^ Hence, it is urgently needed to prepare multifunctional bioimplants that can realize synergistic bone defect reconstruction and tumor recurrence prevention.^15–18^

Currently, traditional metallic implants including stainless steels, cobalt–chromium (Co–Cr) alloys, and titanium (Ti) alloys are commonly employed for repair and reconstruction of bone defects because of their desirable corrosion resistance and mechanical strength.^19–21^ However, these permanent metallic implants cannot degrade in physiological environment after implantation, and a secondary surgical intervention is required to remove these devices after bone healing.^22–25^ The elastic modulus of these permanent metals is much higher than that of natural bone, which often induces the stress shielding effect and results in secondary bone fracture.^26–28^ Moreover, these conventional metallic materials as well as hydroxyapatite bioceramic cannot kill tumor cells and prevent the recurrence of tumors.^29^

Over the past decades, biodegradable magnesium (Mg)-based alloys have drawn much attention in traumatology and orthopedic fields.^30,31^ Owing to their incredible merits including antibacterial activities, biocompatibility, degradability, and good mechanical properties, Mg-based biomaterials are becoming sought-after orthopedic implants.^32–35^ The density of Mg-based alloys (1.74–2 g/cm^3^) is close to that of cortical bone (1.8–2.1 g/cm^3^).^36^ The elastic modulus of Mg-based alloys (20–45 GPa) is also similar to that of human bone (20–27 GPa).^37^ Therefore, Mg alloys possess desirable mechanical properties matching with human bone, which could effectively alleviate the stress shielding phenomenon.^38^ Moreover, Mg alloys are biodegradable osteosynthesis materials and can degrade progressively in the physiological environment and be replaced by new bone finally, obviating the need of secondary surgery to remove residual parts after bone healing, which could effectively reduce medical costs and patient suffering.^39–41^ Many innovative Mg-based products have been developed, including intramedullary nail, scaffold, artificial bandage, etc., which significantly promote new bone formation in the repair of bone defects.^42–45^ Mg ions released from such biomaterials could upregulating the expression of calcitonin gene-related peptide (CGRP) and vascular endothelial growth factor (VEGF), which finally accelerate defect bone regeneration.^46–48^ Recently, it has been indicated that biodegradable Mg alloys exhibited antitumor effects. Kim *et al.* found that Mg and Mg–Ti particles could effectively kill human OCs SAOS_2_ *in vitro* and concluded that Mg–Ti alloys were excellent biomaterials to repair bone defects, resulting from surgical resection and preventing tumor cells from metastasizing.^49^ Zan *et al.* inserted Mg wires into mice with OS, and the Mg wires exhibited satisfactory antitumor performance and effectively suppressed the tumor growth.^29^ Thus, biomedical Mg-based alloys are promising biomaterials for OS patients with large-scale bone destruction generated by surgical intervention. The desired antitumor properties of Mg and its alloys may be caused by their corrosion products, but the exact mechanism remains to be explored.^50,51^

The rapid degradation rate of Mg and its alloys in aqueous environment is a bottleneck that hampers their extensive clinical applications.^52–54^ The fast corrosion speed of Mg metal can be attributed to its high electrochemical activity.^55^ Mg possesses a relatively low standard electrode potential of −2.37 V_SHE_, and, thus, it always acts as the anode and suffers corrosion in contact with other metals,^56^ which is the major reason for its poor corrosion resistance. In addition, the fast degradation speed of Mg alloys will erode their mechanical stability at the early stage and cause implantation failure.^57^ To obtain Mg-based biomaterials with excellent anticorrosion performance, alloying and surface modification are mainly considered.^58–60^ By alloying with Al, Zn, Cu, Ca, Mn, etc., the corrosion performance of Mg-based alloys could be significantly enhanced.^61–65^ The alloying elements at appropriate concentrations can significantly enhance the anticorrosion properties of Mg alloys via reducing the grain size.^66^ Instead, an excessive amount of addition will deteriorate their anticorrosion properties and generate a large number of second phases with potential different to Mg matrix, leading to the aggravated galvanic corrosion of Mg alloys.^67,68^ Meanwhile, given the clinical applications of Mg-based biomaterials, the doping content of alloying elements is also needed to be biosafe and nontoxic. Surface coating aims to prepare protective layers on the surface of Mg alloys, and these layers serve as corrosion barriers to insulate the Mg substrate from the corrosive environment, thus effectively slowing down the corrosion rate of Mg alloys.^69,70^ Generally, surface coating technologies include micro-arc oxidation (MAO),^71^ electroplating,^72^ vapor deposition,^73^ chemical conversion,^74^ etc. In our previous work, we prepared MAO/GelMA hydrogel composite coatings on WE43 alloys, and the experimental results of the immersion and electrochemical tests demonstrated that the corrosion resistance was improved significantly, and the composite coatings exhibited desired cytocompatibility.^75^ However, more efforts are still needed to obtain coatings with tough bonding and multifunctionality on Mg alloys.

As orthopedic implants, biomedical Mg alloys demand a lower corrosion rate to prolong their service time to meet the healing speed of bone, whereas tumor treatments require the opposite strategy.^76^ The enhancement of the anticorrosion properties of Mg-based biomaterials will compromise their antitumor activities. Li *et al.* demonstrated that Mg showed a strong cytotoxic effect on OCs, while the MAO treated Mg exhibited a relatively weak cytotoxic effect because of the reduced corrosion rate.^77^ Therefore, alloying with antitumor metallic elements or introducing therapeutic agents into the coatings on Mg-based alloys can be a novel and useful OS therapeutic option.

In this review, we first discuss the antitumor mechanisms of Mg-based alloys, which are arising from their degradation products. Then, approaches that can simultaneously enhance the anticorrosion properties and antitumor activities of Mg alloys are summarized.

## II. THE ANTITUMOR EFFECTS OF Mg-BASED BIOMATERIALS

More and more researchers have reported the antitumor functions of biomedical Mg-based alloys.^78,79^ Qiao *et al.* confirmed that Mg implants could effectively inhibit the growth of ovarian tumors in mice and induce apoptosis of SKOV3 cells.^80^ Peng *et al.* implanted Mg wires into mouse subcutaneous tumors and verified that Mg-based implants significantly suppressed the growth of gallbladder cancer.^81^ Chen *et al.* reported that metal Mg possessed inhibitory effects on the progression of breast carcinoma *in vivo*.^82^ Antitumor characteristics of Mg-based biomaterials are listed in Table I.

**TABLE I.** Antitumor characteristics of Mg-based biomaterials.

Tumor types	*In vitro* or *in vivo* test	Antitumor factors	Reference
Bone tumor	Both	Mg^2+^	29
Bony cancer	*In vitro*	H_2_	50
Osteosarcoma	*In vitro*	OH^−^	77
Ovarian tumor	Both	Mg^2+^, H_2_	80
Gallbladder cancer	Both	Mg^2+^, OH^−^	81
Breast carcinoma	Both	H_2_	82
Colon carcinoma	Both	H_2_	83
Colorectal tumor	Both	Mg^2+^, H_2_	84
Hepatobiliary carcinoma	Both	Mg^2+^, OH^−^	85

When contact with body fluids in the physiological environment, Mg alloys will be corroded, and the anodic and cathodic corrosion reactions are described later.^86^ The degradation products of Mg alloys including Mg^2+^, OH-, and H_2_ are the source of their antitumor activities,

$$\text{Mg}\;\to \;{\text{Mg}}^{2+}+2e^{-}\;(\text{anodic}\;\text{reaction}),$$ (1)

$$2H_{2}O+2e^{-}\;\to \;2OH^{-}+H_{2}↑\left(\text{cathodic}\;\text{reaction}\right).$$ (2)

### A. The effect of H_2_

As a safe endogenous gas, the antitumor effects of H_2_ have been reported.^87–89^ The first report of H_2_ as a therapeutic agent for tumors was carried out by Dole *et al.* in 1975, they treated skin squamous cell carcinoma via hyperbaric H_2_, and the tumor growth of mice was effectively inhibited.^90^ In 2007, Ohsawa and colleagues discovered that H_2_ could selectively scavenge reactive oxygen species (ROS) including peroxynitrite (ONOO^−^) and hydroxyl radicals (•OH).^91^ The ROS, especially •OH, plays a vital role in the occurrence and metastasis of tumors, and such role could be significantly restrained after the ROS was quenched.^92,93^ Thus, Mg alloys may be ideal biomaterials for OS patients with extensive bone defects due to the released H_2_ during their degradation. Qiao *et al.* confirmed that the released H_2_ from Mg degradation induced the apoptosis of ovarian tumor cells through reducing the amount of ROS inside tumor cells.^80^ It is reported that the antitumor effect is proportional to the speed of H_2_ release during Mg degradation.^50^

**FIG. 1.**
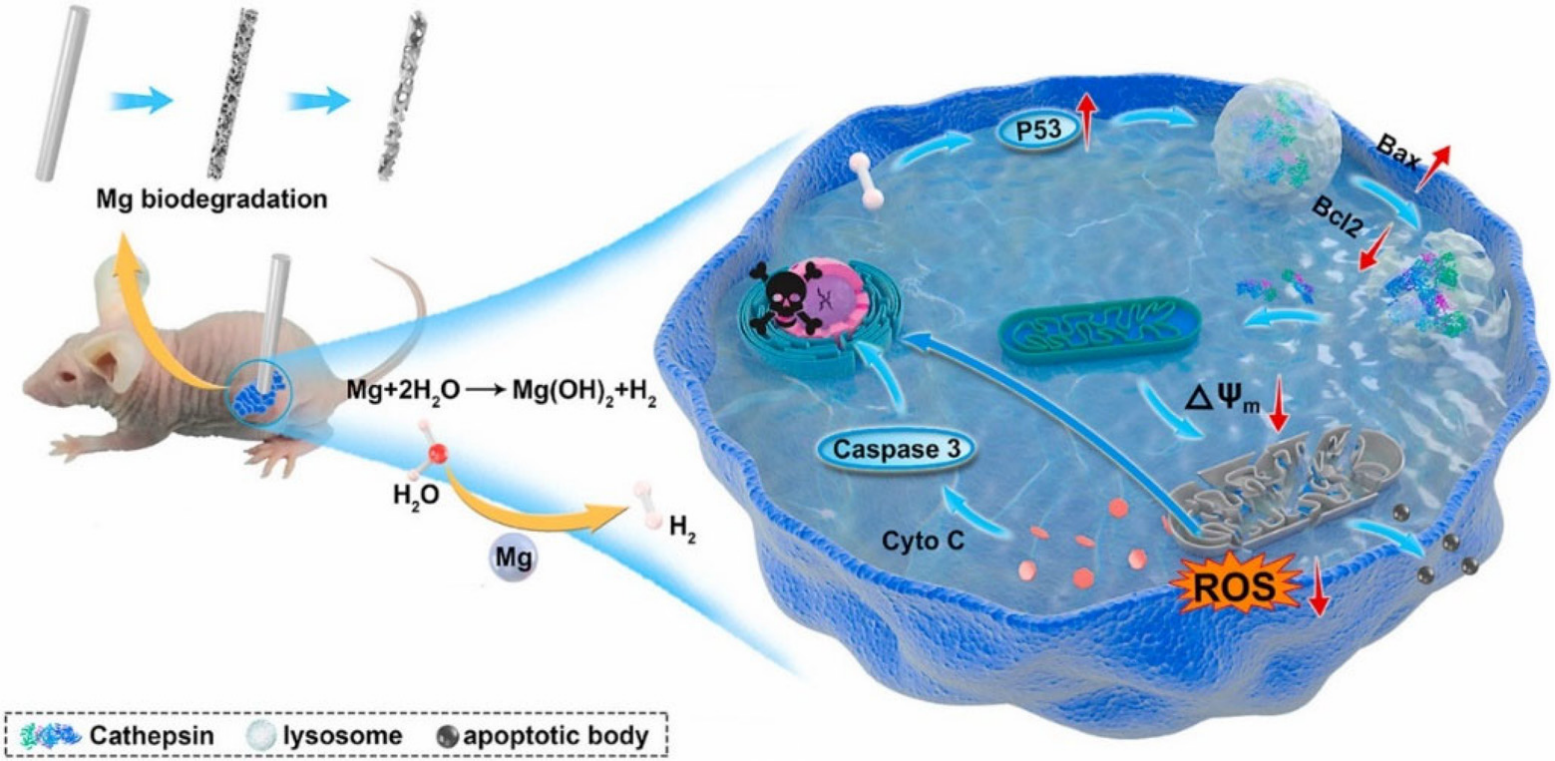
Schematic diagram of the antitumor effect of H_2_ from biodegradable Mg.^83^ Reproduced with permission from Zan *et al.*, Bioact. Mater. **9**, 385–396 (2022). Copyright 2018 Authors, licensed under a Creative Commons Attribution (CC BY) license.

In their recent work, Yang *et al.* developed Mg-based galvanic cell rods, which were implanted into tumors in mice subsequently, and they demonstrated that the continuous generation of H_2_ could reduce the membrane potential of mitochondrial, affecting the synthesis of ATP and destructing the intracellular balance of redox in tumor cells, which finally suppressed tumor cell respiration and significantly inhibited the growth of tumor.^94^ In addition, there is another potential antitumor mechanism of H_2_. Zan *et al.* proved that the sustained release of H_2_ could upregulate the expression of P53, which was a tumor suppressor protein. After that, the P53 proteins triggered the rupture of lysosome to release cathepsin B, which then lowered the membrane potential of mitochondria. Then, the mitochondrial membrane would be damaged, leading to an obvious decrease in the level of ROS and the release of pro-apoptotic protein cytochrome C as well as apoptotic body, which eventually induces the apoptosis of tumor cells. The described antitumor mechanism of H_2_ is demonstrated in Fig. 1.^83^ However, it is necessary to ensure that H_2_ generated from the degradation of Mg alloys has specific cytotoxicity on tumor cells, while it has no effect on the integrity of adjacent normal cells. Generally, tumor cells have higher levels of ROS than healthy cells, and tumor cells are more sensitive to the changes of ROS level.^95^ As a selective antioxidant, H_2_ can eliminate the ROS in tumor cells and thereby exerting inhibitory effect on the growth of tumor cells, but it has no effects on the role of ROS in healthy cells.^29,96^ Therefore, the strategy of using Mg alloys to eliminate residual tumor cells of OS patients after surgery may benefit from a higher tumor cell sensitivity toward H_2_ compared with adjacent normal cells.^76,97^

### B. The effect of Mg^2+^

Mg element participates in several physiological activities, exhibiting excellent osteogenic effects and can stimulate the formation of new bone.^44^ It is reported that Mg^2+^ could also act as an agent for the prevention and therapy of OS.^98^ Qiao *et al.* demonstrated that Mg^2+^ with a concentration exceeding 20 mM could significantly inhibit the proliferation and promote the apoptosis of ovarian cells. They speculated that Mg^2+^ suppresses the growth of ovarian cells through blocking the cell cycles in the G0/G1 stage.^80^ Peng *et al.* also confirmed that Mg^2+^ at a concentration higher than 30 mM could suppress the growth of gallbladder cancer cells and trigger their apoptosis, while they reported that the number of cancer cells at the G0/G1 cultured in the medium containing Mg^2+^ was less than that of the control group (p < 0.05), which can be attributed to the fact that Mg^2+^ promotes the synthesis of DNA during the early division phase of cancer cells.^81^ Therefore, more work is needed to clarify such seemingly contradictory results of Mg^2+^ on the cell cycles of tumor cells.

**FIG. 2.**
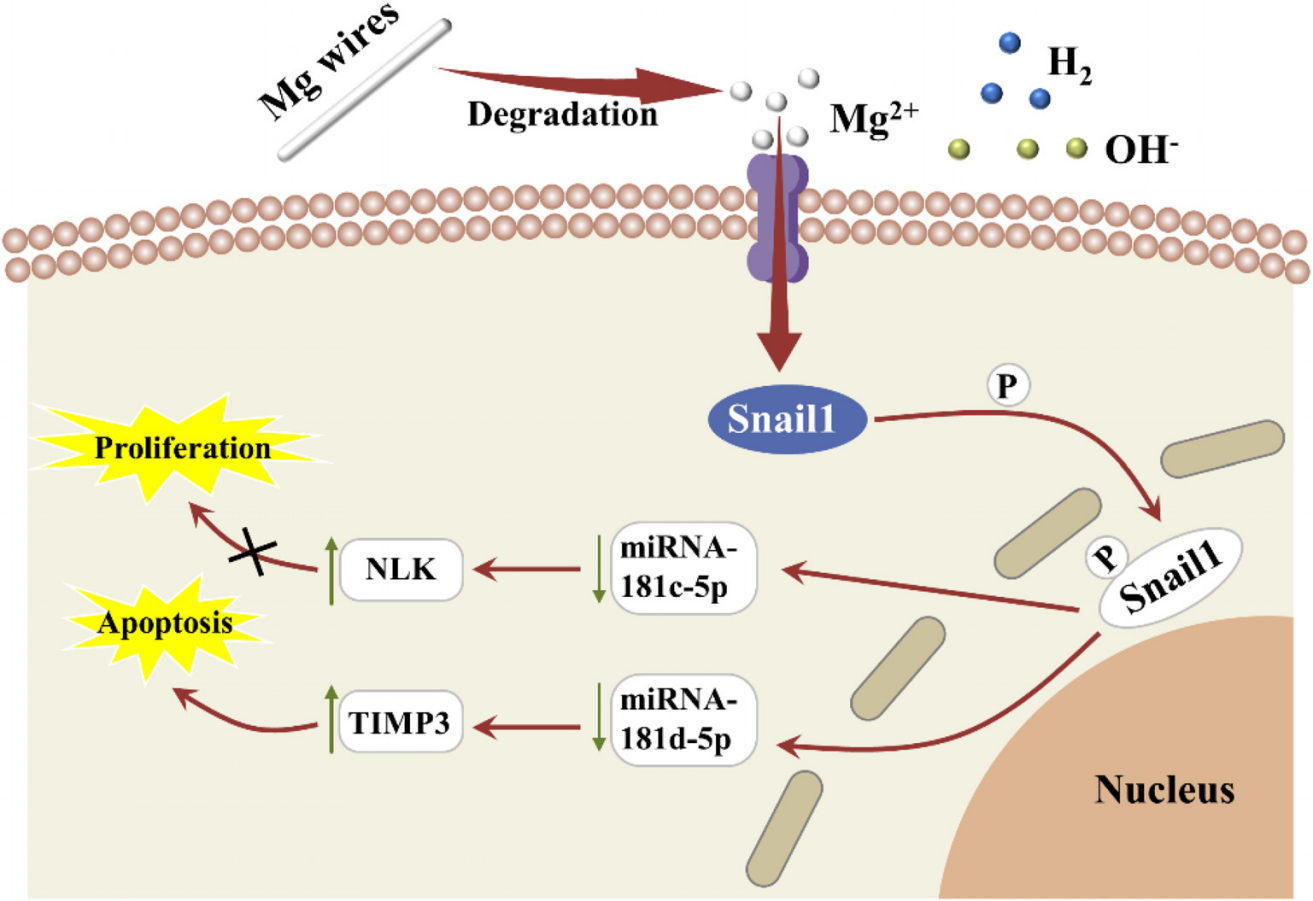
Schematic illustration of a potential antitumor effect of Mg^2+^ from biodegradable Mg.

In addition, other researchers have also elaborated on the antitumor mechanism of Mg^2+^ from different perspectives. Wei *et al.* found that Mg^2+^ released from the degradation of Mg coating could induce autophagy-dependent apoptosis through the AMPK/mTOR/ULK1 pathway.^51^ In addition, Zan *et al.* proposed a potential Mg^2+^-mediated signaling pathway for the suppression of OCs. They reported that the excessive Mg^2+^ produced by the degradation of Mg wires was transported into tumor cells via the TRPM7 channel and led to the phosphorylation of Snail1 protein, which was subsequently imported back to the nucleus. The phosphorylated Snail1 protein in the nucleus could effectively lower the level of miRNA-181c/d-5p, which then activated the expression of TIMP3 and NLK proteins and, finally, suppressed the proliferation, migration, and invasion of OCs.^29^ The described antitumor mechanism of Mg^2+^ is shown in Fig. 2. However, Zhang *et al.* independently studied the influence of Mg^2+^ on the OCs and found that the increase in Mg^2+^ did not kill the OCs.^99^ Hence, there is still more work to be done to elucidate the antitumor mechanism of Mg^2+^.

### C. The effect of pH value

The tumor microenvironment was reported to be acidic with a pH range from 5.7 to 7.0, which was beneficial for the survival of tumor cells and played a significant role in the growth and progression of tumors.^100,101^ The acidic tumor microenvironment was caused by the accumulation of lactate produced from their high rate of aerobic glycolysis.^102^ Thus, the increase in local pH surrounding Mg-based implants caused by the released OH- from their degradation would suppress the proliferation and metastasis of the remaining tumor cells around the bone defects.^103^ Zhang *et al.* confirmed that the rise of pH value resulting from the corrosion of Mg showed a strong cytotoxic effect on OCs.^99^ The influence of pH value on different tumor cells is summarized in Table II, which reveals that diverse types of tumor cells exhibit different tolerance to pH value, while a higher one is inclined to promote the apoptosis of tumor cells.

**TABLE II.** The influence of pH value on different tumor cells.

Tumor cell types	pH value	Effect	Reference
Osteosarcoma cells MG-63	7.4–8.0	Without negative effect	29
Ovarian cancer cell SKOV3	7.4–8.0	Fail to affect	80
	8.3	Induce apoptosis	
Gallbladder cancer cells SGC-996	7.5	Do not affect	81
	≥7.8	Significantly inhibit	
Osteosarcoma cells U2-OS	9.43	Begin to lose integrities	99
	> 10	Do not clearly exist	

How does the high alkalinity achieve its inhibitory effect on the growth of tumor cells? Li *et al.* reported that bare Mg and MAO coated Mg samples exhibited a negative effect on the adhesion of OCs because of the rise of pH value during their corrosion. They supposed that the alkaline microenvironment could destroy the cytoskeleton F-actin in OCs, which was very important for the regulation of tumor progression and growth.^77^ By adjusting the pH values of the cell culture medium with NaHCO_3_ solution, Peng *et al.* discovered that the synthesis of DNA would be hindered in the alkaline environment, leading to an increase in the number of cells at the G0/G1 stage and eventually suppressing the growth of SGC-996 cells.^81^ Li *et al.* speculated that the elevation of the pH value resulting from Mg degradation could suppress the expression of HIF-1α and its downstream protein CAIX, which, finally, suppressed the growth of tumor cells and induced their apoptosis. However, the influence of Mg^2+^ and the released H_2_ cannot be ignored because they cocultured tumor cells with Mg leaching solution.^85^

#### 1. Mechanism based on antitumor immunity

**FIG. 3.**
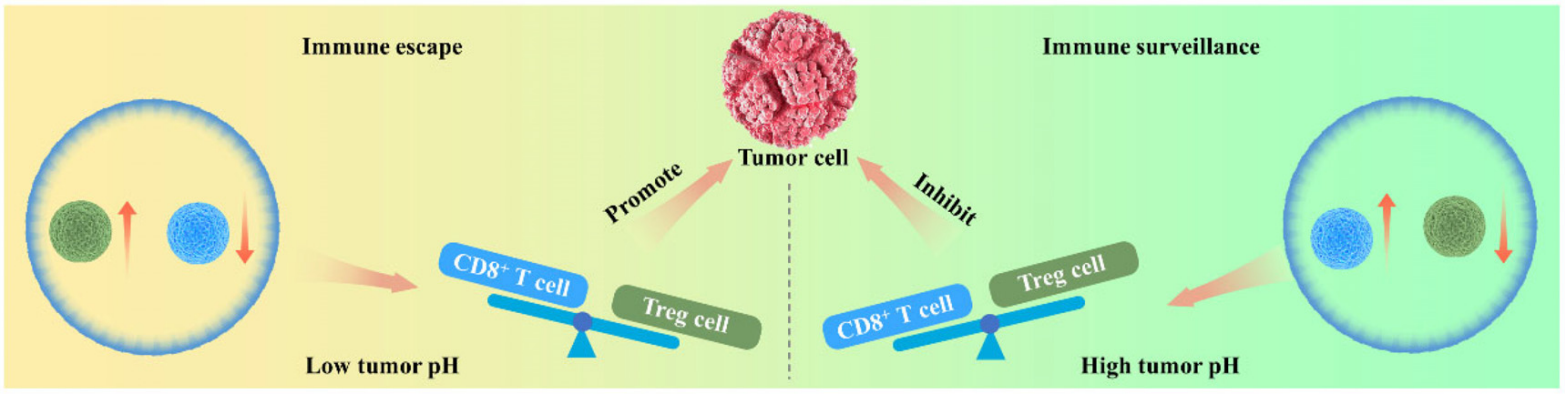
Schematic drawing of the antitumor effect of alkalinity via antitumor immunity.

Moreover, there is a potential antitumor mechanism of alkalinity related to antitumor immunity. Although the CD8^+^ T cells play a vital role in modulating the progression of tumors because of their ability to kill malignant cells,^104^ they are inhibited and impotent in the acidic tumor microenvironment.^105–107^ It was believed that the immune suppressive regulatory T (Treg) cells, which were activated by the tumor acidity, could also blunt CD8^+^ T cells.^108–110^ Therefore, by antagonizing the tumor acidity, the CD8^+^ T cells can be activated, while the Treg cells are suppressed, which will turn the immune escape of tumor cells to immune surveillance and, therefore, help inhibit the growth of tumor.^111^ Hence, the alkaline microenvironment caused by the corrosion of Mg alloys can suppress the growth of tumors through antitumor immunity. The discussed process of antitumor of alkalinity through antitumor immunity is described in Fig. 3.

#### 2. Mechanism based on oxidative stress

**FIG. 4.**
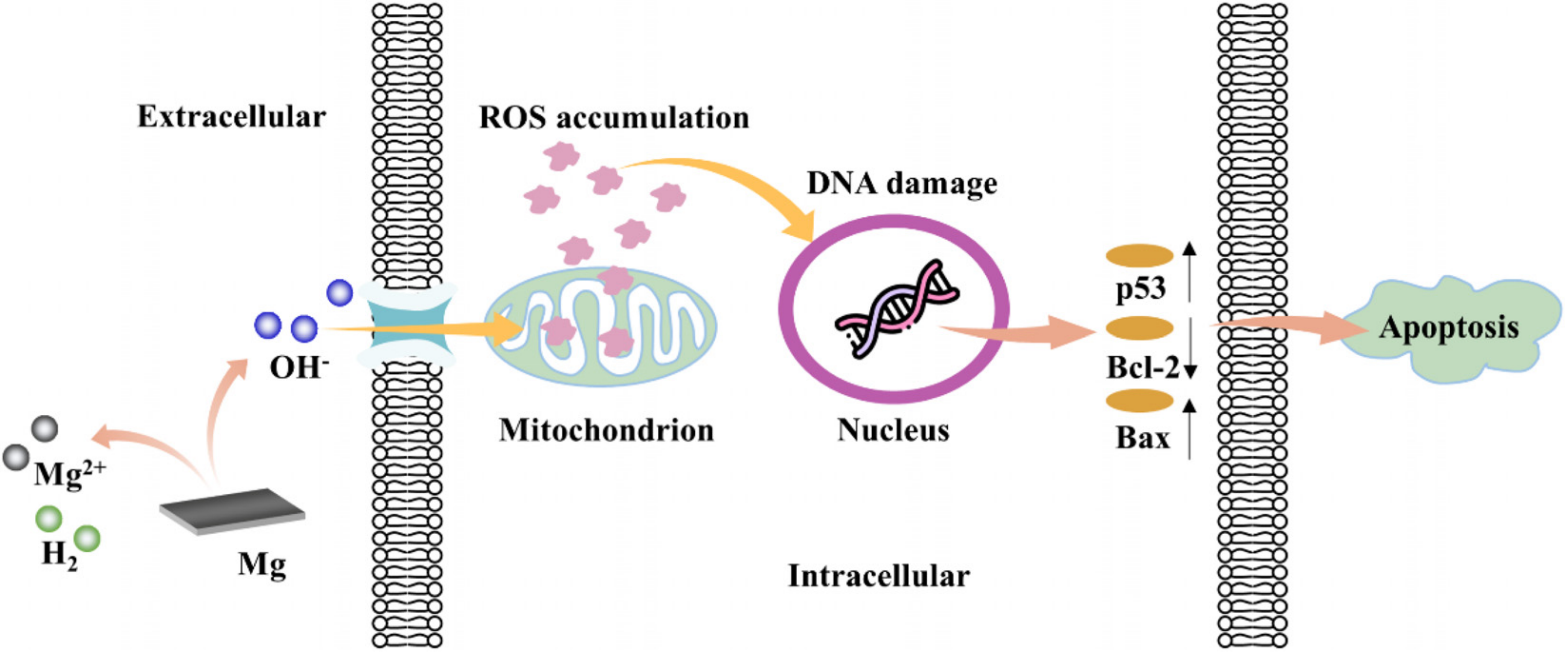
Schematic drawing of the antitumor effect of OH^−^ from biodegradable Mg.

In addition, another interesting antitumor mechanism of OH- is also been explored. The corrosion of Mg-based alloys can generate OH- and increase the pH value, and it was found that a high pH value could inhibit the functions of superoxide dismutase (SOD), peroxidase, and catalase.^112^ These enzymes are usually responsible for the cellular antioxidant defenses by scavenging ROS, and the suppression of such enzymes can result in the accumulation of ROS within tumor cells.^113^ The excessive ROS in the tumor cells can interact with DNA and result in oxidative DNA damage,^114^ which then triggers the expression of the p53 protein.^115^ As one of the crucial tumor suppressors, p53 finally induces the apoptosis of tumor cells.^116^ In addition, the activated p53 can subsequently upregulate the expression of Bax while down-regulate that of Bal-2, and the imbalance of Bax/Bcl-2 can also lead to tumor cell apoptosis.^117,118^ This antitumor mechanism of OH^−^ is depicted in Fig. 4. The tumor-promoting function of ROS in Sec. II A seems to contradict this tumor-suppressing function, and the relationship between ROS and tumor development is still controversial.^119^ The corrosion products of Mg alloys may exert diverse effects on the ROS, and different ROSs possess very different targets and activities. Therefore, the antitumor mechanisms of Mg alloys through ROS may involve synergistic effects of multiple factors, and more innovative and systematic investigations are still required to elucidate the specific situation.

## III. STRATEGIES TO IMPROVE THE ANTICORROSION AND ANTITUMOR EFFECTS SIMULTANEOUSLY

According to the aforesaid, biomedical Mg metals exhibit great potential in suppressing the recurrence and metastasis of tumors due to their degradation products. However, it was reported that after being inserted into the body, a uniform Ca–P film will be formed on the surface of Mg and serve as a protective layer to slow down its degradation.^80^ In addition, the existing buffering system of the human body can partially counteract the alkalinity caused by the degradation of Mg metals.^120^ Thus, the antitumor effects of Mg metals may not be maintained *in vivo* and need to be enhanced. However, the fast corrosion rate of Mg metals may lead to mechanical integrity loss at an early stage before the completion of bone defect reconstruction, which will lead to the failure of surgery.^121^ Thus, alloying with antitumor elements or coating with antitumor layers may be useful strategies to simultaneously enhance the anticorrosion and antitumor effects of Mg-based implants and facilitate the advancement of such innovative medical devices to repair bone defects caused by tumor curettage.

### A. Alloying with antitumor metallic elements

Experiments *in vitro* have revealed that alloying with elements such as rare earth (RE), silver (Ag), or zinc (Zn) could endow Mg alloys with significant inhibitory effects on diverse tumor cells.^117,122,123^ Hence, although the relevant literature is limited, we still have reasons to believe that alloying Mg with these bioactive elements will simultaneously improve the anticorrosion properties and the antitumor activities of Mg alloys.

#### 1. Alloying with RE elements

**FIG. 5.**
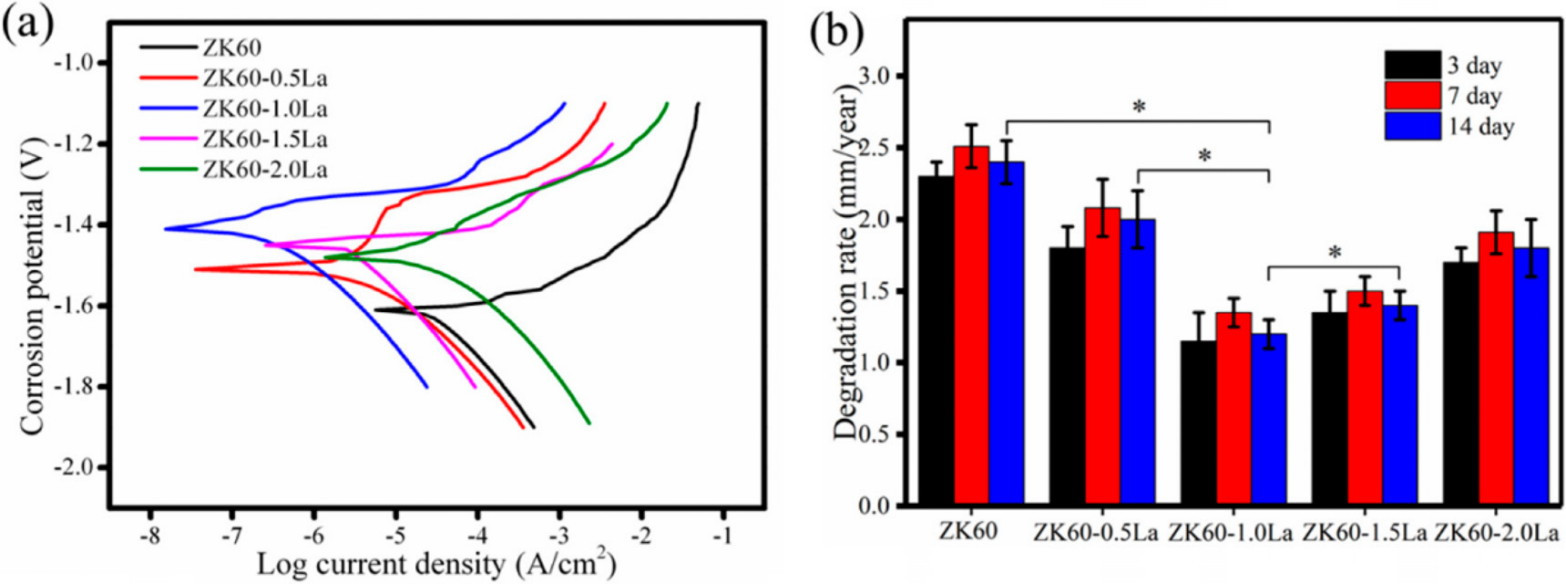
(a) Electrochemical tests of ZK60 and ZK60-xLa and (b) degradation rates of ZK60 and ZK60-xLa.^125^ Reproduced with permission from Shuai *et al.*, Appl. Sci.-Basel **8**, 2109 (2018). Copyright 2018 Authors, licensed under a Creative Commons Attribution (CC BY) license.

It was reported that RE elements could boost the antitumor activities of Mg-based biomaterials, and Mg alloys containing RE elements are regarded as promising implants in orthopedic oncology.^124^ Shuai *et al.* alloyed the ZK60 Mg alloy with Lanthanum (La) by selective laser melting and prepared ZK60-xLa (x = 0, 0.5, 1.0, 1.5, and 2.0) alloys. Their results revealed that ZK60-1.0La alloy exhibited a significant suppression effect on the growth of bone tumor cells and excellent biocompatibility for healthy cells compared with the ZK60 group, which was mainly due to the released La ions and the high alkalinity resulted from the corrosion of Mg alloy.^125^ Due to a higher ROS level, tumor cells are more vulnerable to oxidative stress compared to healthy cells.^95^ La ion with a greater ratio of electric charge to ion radius can easily bind with divalent metal on mitochondria, leading to the open of mitochondrial permeability transition pore, which can result in the block of the electron transport chain in mitochondrial and cause the generation and accumulation of ROS.^126–129^ The increased level of ROS eventually triggers tumor cell apoptosis through a high oxidative stress.^130–132^ In addition, the corrosion rate of ZK60-1.0La is 1.23 mm/year, which was much less than that of the 2.13 mm/year of the ZK60 alloy, as shown in Fig. 5, which was primarily because of the grain refinement. By carefully controlling the doping content, Anisimova *et al.* found a balance between the anticorrosion properties and the antitumor effect of Mg-10%Gd alloy, and they confirmed that the antitumor properties could be attributed to the released Gd^2+^ ions during the biodegradation of Mg-10%Gd alloy.^123^ Meanwhile, they also studied the antitumor features of WE43 alloy on MDA-MB-231 as well as LNCaP tumor cells, which often lead to bone metastasis. They found that the coincubation of WE43 alloy with such two kinds of tumor cells inhibited their growth and induced apoptosis, which could be attributed to the released RE elements and the elevated alkalinity caused by the degradation of Mg alloy.^124^

#### 2. Alloying with Ag element

**FIG. 6.**
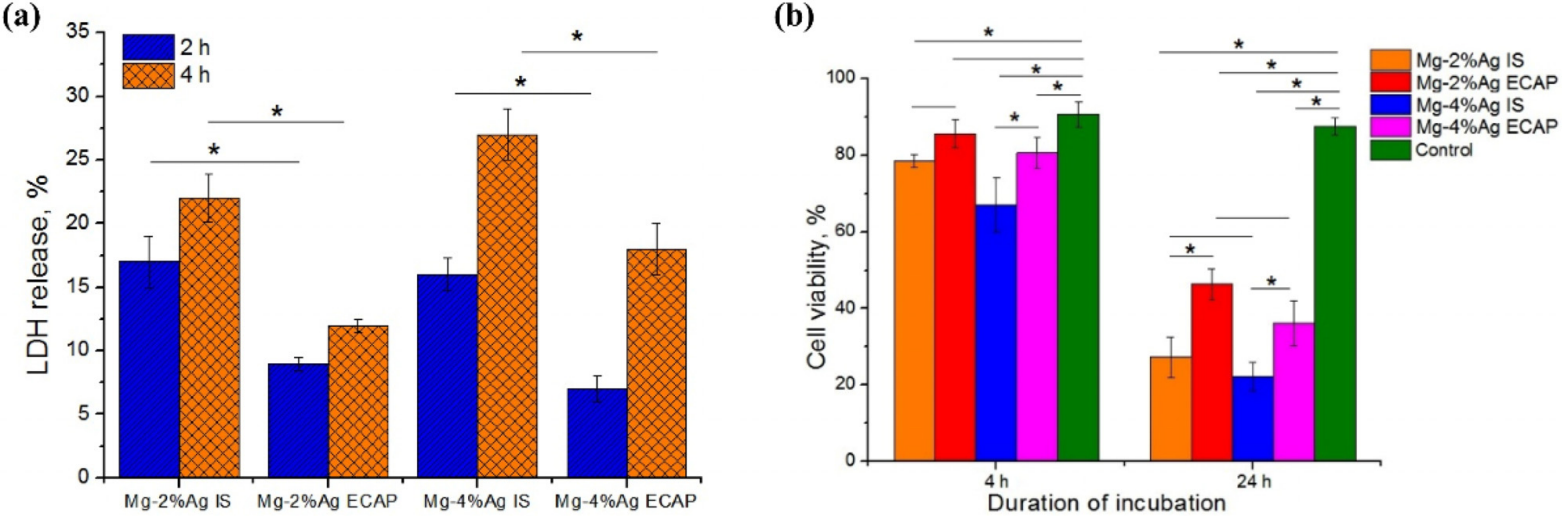
(a) The LDH release as a function of different Mg–Ag alloys at different states (initial state (IS) and equal-channel angular pressing state (ECAP)) after 2 and 4 h of incubation; (b) effect of diverse Mg–Ag alloys on the viability of tumor cells.^140^ Reproduced with permission from Estrin *et al.*, Materials **12**, 3832 (2019). Copyright 2018 Authors, licensed under a Creative Commons Attribution (CC BY) license.

The antibacterial functions of Ag element have been widely accepted,^133–135^ and its antitumor attributes have also received increasing attention.^136–138^ Satapathy *et al.* demonstrated that Ag-based nanoparticles (AgNPs) caused the apoptosis of human colon carcinoma cells in a p53-dependent manner. The presence of AgNps results in an upregulation of the tumor suppressor gene p53. Activation of p53, on the one hand, enhances the expression of its downstream target gene p21, thereby facilitating DNA damage and, on the other hand, reduces the levels of anti-apoptotic markers NF-κB and AKT. Furthermore, activation of p53 also promotes BAX/BCL-XL expression and augments caspase cleavage. Collectively, these events ultimately induce apoptosis in tumor cells.^139^ Accordingly, Mg–Ag alloys can not only prevent infections associated with OS after tumor resection but can also suppress the growth and metastasis of tumor.^76^ Estrin *et al.* cocultured Mg–Ag alloys with human leukemia cells and observed that the Mg alloy with a higher content of Ag resulted in an increased level of lactate dehydrogenase (LDH) in the solution and a stronger cytotoxic effect on the tumor cells as presented in Fig. 6, which manifested the potential of Mg–Ag alloys as orthopedic implants in clinical oncology.^140^ Although there are a few descriptions and discussions on the corrosion behaviors of Mg alloys in the listed literature, the strategy of obtaining Mg-based implants with anticorrosion and antitumor properties by alloying is feasible.^135^ It is vital to find an optimal degradation rate of Mg–Ag alloys to obtain stronger antitumor effects while maintaining the mechanical integrity of implants at the early stage of implantation as well as exerting negligible cytotoxic effect toward healthy cells. It is suggested that the optimal degradation rate of Mg–Ag alloys is in the range of 1.5–2.2 mm/year.^141^ What in needed to be done in the future is to determine the suitable doped content of Ag to enhance the anticorrosion properties and antitumor effects of Mg alloys as well as their biocompatibility.

#### 3. Alloying with Zn element

**FIG. 7.**
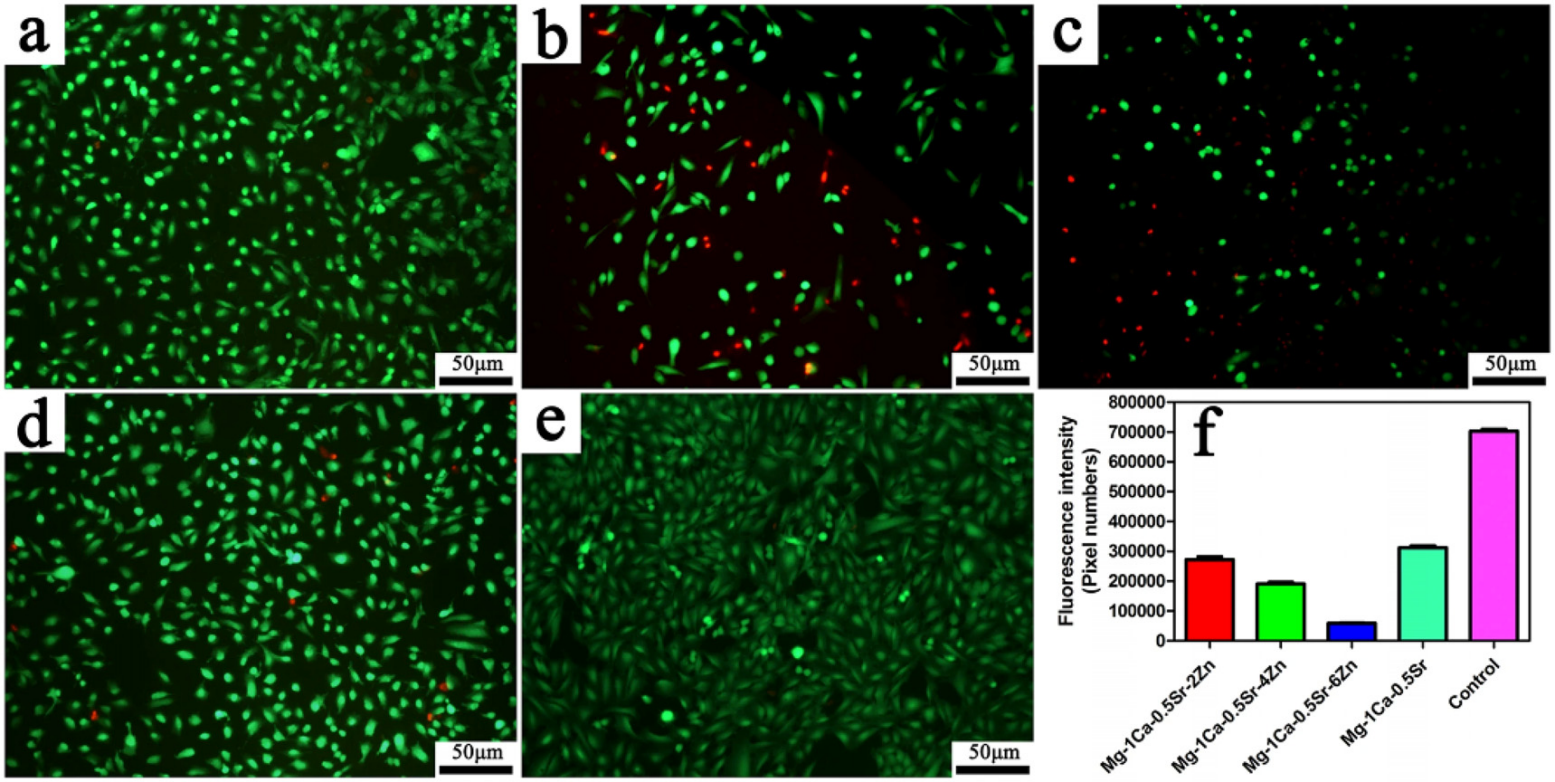
Live/dead staining of the U2OS cells cocultured with the extracts of Mg-1Ca-0.5Sr-xZn after 5 days, green means the live cells, and red presents dead cells. (a) x = 2; (b) x = 4; (c) x = 6; (d) x = 0; (e) Ti-6Al-4V control; and (f) fluorescent intensity analysis of the live cells in different extracts.^117^ Reproduced with permission from Wu *et al.*, Sci. Rep. **6**, 21736 (2016). Copyright 2018 Authors, licensed under a Creative Commons Attribution (CC BY) license.

Zn participates in many physiological activities and plays a vital role in human health,^142^ and it is reported that Zn could efficiently induce the apoptosis of human alveolar adenocarcinoma cells.^143^ The accumulation of Zn ions in tumor cells can lead to the breakdown of mitochondrial transmembrane electrochemical gradient, leading to the increased level of ROS, which, finally, triggers the apoptosis of tumor cells via oxidative stress.^144–146^ Such inhibitory effect is selectively targeted to tumor cells and exhibits negligible cytotoxicity on normal cells. Taken together with its excellent antibacterial performance,^147^ Mg–Zn alloys are expected to be another promising bone implant for OS patients. Wu *et al.* conducted a series of experiments and found that the extracts of Mg alloys containing diverse amounts of Zn obviously suppressed the growth and proliferation of U2OS cells *in vitro*, and the inhibitory effects were proportional to Zn contents in Mg alloys (Fig. 7). Although the alkaline microenvironment resulted from the corrosion of Mg alloys may have some impact, they confirmed that Zn^2+^ in the extracts was the main factor for the antitumor behavior.^117^ Based on their results, Mg alloy containing 6 wt. % Zn would be an ideal orthopedic implant for bone defect reconstruction in OS patients. While ensuring the antitumor effects, attention should also be paid to the anticorrosion properties of Mg-Zn alloys. By carefully controlling the doping content of Zn, the anticorrosion performance of Mg alloys can be obviously enhanced.^148,149^

### B. Surface modification with antitumor coatings

Another strategy to balance the antitumor effects and the anticorrosion properties of Mg alloys are surface modification with antitumor coatings. Such antitumor coatings can endow Mg alloys with excellent antitumor activities as well as enhanced corrosion resistance, which is bound to overcome the challenges in orthopedic oncology.^150^ Here, three types of antitumor coatings including antitumor drugs-loaded coatings and coatings with external field response effects as well as coatings with inherent antitumor properties are introduced as follows.

#### 1. Antitumor drug-loaded coatings

Drugs such as paclitaxel (PTX), doxorubicin (DOX), and cisplatin have been widely used in cancer therapy,^151–153^ but the systemic side effects caused by the antitumor agents still cannot be neglected.^154^ Hence, Mg alloys loaded with antitumor agents may be a promising solution to this dilemma. Mg-based implants can serve as drug delivery vehicles to directly release therapeutic agents and target the tumors, which can effectively eradicate the remaining OCs and inhibit tumor recurrence after OS surgery while reducing the side effects of systemic administration.^155,156^

**FIG. 8.**
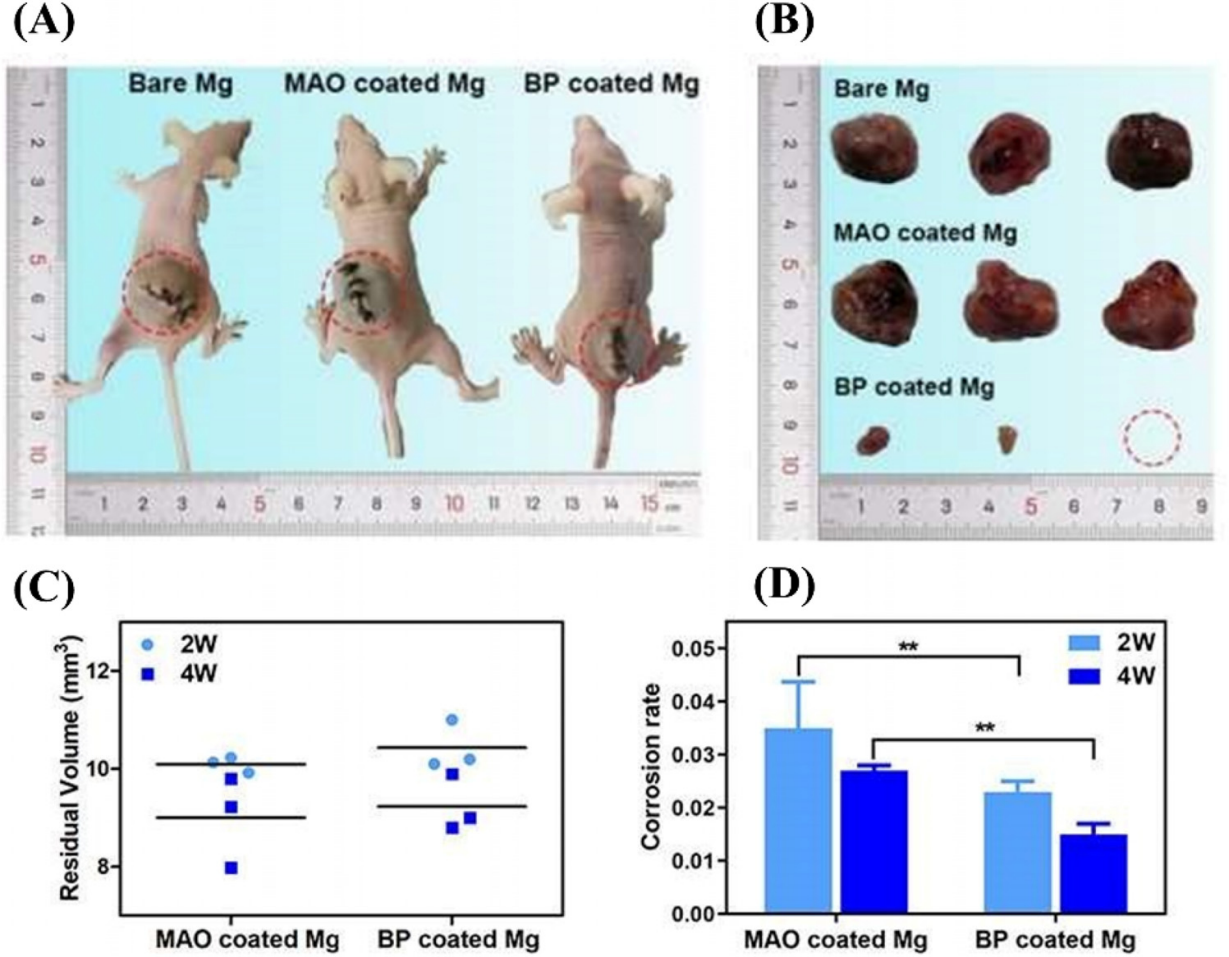
(a) Pictures of three groups of mice inserted with Mg pellets at the tumor sites after 4 weeks. (b) Different tumor tissues of mice inserted with diverse samples after 4 weeks. (C) Residual volumes of Mg-based implants after 2 and 4 weeks. (D) Corrosion rate of different Mg-based materials.^161^ Reproduced with permission from Li *et al.*, Acta Biomater. **121**, 682–694 (2021). Copyright 2021 Elsevier.

Celastrol, a promising and useful Chinese medicine, has been extensively investigated in clinics.^157^ It can induce the apoptosis and autophagy of tumor cells through activating the ROS/JNK signaling and halting the Akt/mTOR signaling pathway.^158^ Furthermore, it also possesses the ability to block the progression of cell division and the metastasis of tumor cells, thereby exerting its antitumor activities.^159^ Cheng *et al.* sealed the surface of AZ31 Mg alloys by layered double hydroxide (LDH) coatings loading with celastrol through hydrothermal treated and subsequently immersed in celastrol solution, and the prepared coating provided a strong corrosion protecting performance for Mg alloy. Moreover, the developed Mg alloy exhibited a strong inhibitory effect on cancer cells because of the sustained release of celastrol while exerting little negative effects on healthy cells.^160^ Li *et al.* prepared a bisphosphonate (BP)-loaded MAO layer on the Mg–Sr alloy pellet (denoted as BP-coated Mg) through immersing the MAO treated Mg alloy in the zoledronic acid (ZA) solution. The *in vitro* experiments verified that the prepared BP-coated Mg could promote the apoptosis and necrosis of OCs as well as prevent their invasion. More importantly, the *in vivo* implantation tests also demonstrated that the developed BP-coated Mg obviously suppressed tumor growth, and the prepared double layer effectively enhanced the anticorrosion properties of Mg–Sr alloy pellet, as shown in Fig. 8. The antitumor functions of the BP-coated Mg were attributed to the synergistic effect of the degradation of Mg–Sr alloy and the sustained release of the loaded drug.^161^ As a nitrogen-containing BP, ZA shows desirable therapeutic effect on the primary bone tumors. Antitumor drug-loaded coatings on Mg alloys directly target the remaining OCs around the bone defects with a high local concentration drug release, which can largely reduce the serious side effects and improve the prognosis of OS patients.

#### 2. Antitumor coatings with external field response effects

Materials that can respond to the optic, magnetic, or acoustic energy and produce thermal energy exhibit great potentiation in tumor therapy.^162–165^ The generated local tissue hyperthermia can effectively induce cancer cell death and tumor remission. Coatings with such external field response effects can be fabricated on the Mg alloys and realize the precise treatment of OS and avoid drug resistance.

Photothermal therapy (PPT) is a typic external field response therapy, and it utilizes the photothermal property of materials and converts the optic energy of near infrared irradiation (NIR) into thermal energy, which can efficiently kill tumor cells.^166^ Du *et al.* used Mg–Fe LDH as a precursor and prepared ferric oxide (Fe_3_O_4_) nanosheets on plasma electrolytic oxidation (PEO) treated Mg alloys, and the prepared Mg alloys exhibited strong anticorrosion properties and desirable biocompatibility. Furthermore, the Fe_3_O_4_ nanosheets exhibited effective antitumor activities *in vitro* and *in vivo*, which can be attributed to their good photothermal/chemodynamic properties.^167^ Zhang *et al.* designed a novel LDH coating composed of Fe-rich top and Mn-rich bottom (LDH-MnFe). The fabricated bilayer significantly inhibited the progression of tumors under NIR. It also improved the anticorrosion properties and the biocompatibility of Mg alloys.^168^ Coatings with external field response effects endow Mg alloys with intelligent antitumor abilities, which can precisely eradicate the residual OCs as well as prevent the recurrence and metastasis of tumors. In addition, such coatings also enhance the anticorrosion performance of Mg alloys. Therefore, Mg alloys coated with external field response films may have a bright future in the bone defect reconstruction resulting from tumor reaction. However, much work is still required to be done to explore the strategies of preparing magnetic or acoustic energy response coatings on Mg alloys for the treatment of OS.

#### 3. Coatings with inherent antitumor properties

In addition to the antitumor drugs-loaded coatings and the antitumor coatings that need external stimulus, coatings with inherent antitumor properties may be another good choice. RE-based conversion coatings (RECCs) can provide desirable corrosion protection for the underlying metals.^169^ The antitumor effects of RE elements have been reported.^170–172^ Therefore, preparing RECCs on the Mg alloys will grant Mg-based biomaterials with improved anticorrosion properties and antitumor activities, which can expand the applications of Mg alloys in bone repair. Kannan *et al.* prepared a samarium oxide film on the surface of Mg alloy by electrophoretic deposition. They reported that the samarium oxide-coated Mg alloys exhibited intriguing antitumor effects as well as enhanced anticorrosion properties compared to the naked Mg alloy. In addition, the prepared Mg alloys also exhibited inherent antibacterial activities against *Escherichia coli* (*E. coli*) and *Staphylococcus aureus* (*S. aureus*).^173^ The cytochrome P450 in the OCs is very important for the controlling of tumor microenvironment and tumorigenesis.^174,175^ Samarium ions can specifically interact with two charged side chains of the P450 and inhibit its activity, which finally leads to the death of tumor cells.^176^ More types of RECCs with desirable antitumor and anticorrosion properties are still need to be explored for the treatment of OS.

## IV. CONCLUSIONS

In this presented review, the potential antitumor mechanisms of Mg alloys are proposed, which originate from their corrosion products including H_2_, Mg^2+^, and OH-. Considering the biocompatibility, biodegradability, and antitumor properties, Mg-based orthopedic implants are promising candidates for OS patients after tumor removed. In addition, approaches to balance the anticorrosion properties and antitumor activities are also summarized. Through alloying with antitumor metallic elements such as RE, Ag, and Zn, Mg alloys can be endowed with better anticorrosion properties and antitumor activities. It should be noted that the doping content of these antitumor elements should be carefully controlled to generate better antitumor effects as well as maintaining biocompatibility while avoiding galvanic corrosion because of the formation of the second phase. Another feasible strategy is surface modification with antitumor coatings. While the antitumor drug-loaded coatings allow target delivery and precise treatment, the limited drug loading amount, drug resistance, and uncontrollable drug release are still challenging.

To promote the applications of Mg-based biomaterials in orthopedic oncology, much attention should be paid to determining the effect of ROS produced by Mg degradation on OS development. It is necessary to explore the *in vivo* biosafety of these antitumor alloying elements and explore their proper concentration to inhibit the growth of neoplastic cells without influencing normal cells. While Mg-based biomaterials as bone substitutes need low degradation rate to support the healing of bone defects and avoid significant cytotoxic effect on the adjacent tissues, tumor therapy demands opposite strategies. Therefore, it is very important to tailor the degradation rate of Mg-based implants to ensure the balance of corrosion resistance and the antitumor effects. Attention also needed to be paid to the design and preparation of innovative and intelligent coatings with long-term antitumor and antibacterial properties. In addition to the corrosion resistance and antitumor effects, attention should also be paid to the osteogenic and angiogenic activities of Mg-based implants, as these properties are very important for bone defect reconstruction of OS patients after surgical resection. As one of the implanted medical devices, clinical trials of antitumor Mg-based alloys are required for the safety and effectiveness assessment before entering the marketplace, and strict post-marketing surveillance should also be established. The green approval pathway of antitumor Mg-based implants will facilitate their clinical applications and thereby bringing new treatment hope to OS patients.

## Data Availability

The data that support the findings of this study are available from the corresponding authors upon reasonable request.
